# Breaking down barriers: rationalisations and motivation to stop among Chinese male smokers under cigarette dependence

**DOI:** 10.1186/s12889-024-19295-y

**Published:** 2024-07-08

**Authors:** Dan Zhang, Wen-jiao Chen, Xiao-xia Meng, Xiong Zhao, Run-hua Liu, Hai-yu Tian

**Affiliations:** 1 School of Medicine and Health Management, Guizhou Medical University, No. 6 Ankang Avenue, Guiyang City, 561113 China; 2 School of Sociology, Guizhou Minzu University, Guiyang, China; 3 Center of Medicine Economics and Management Research, Guizhou Medical University, Guiyang, China; 4 Guizhou Institute of Health Development, Guizhou Medical University, Guiyang, China; 5 One Health Institute, Guizhou Medical University, Guiyang, China

**Keywords:** Smoking rationalisation beliefs, Motivation to stop smoking, Cigarette dependence, Partial least squares structural equation modelling, Multi-group analysis, Importance-performance map analysis

## Abstract

**Background:**

Smoking rationalisation beliefs are a huge barrier to quitting smoking. What types of rationalisations should be emphasised in smoking cessation interventions? Although past literature has confirmed the negative relationship between those beliefs and motivation to stop smoking, little is known regarding the importance and performance of those beliefs on motivation with varying cigarette dependence. The study aimed to ascertain rationalisations that are highly important for motivation yet perform poorly in different cigarette dependence groups.

**Methods:**

The cross-sectional study was conducted from November 19 to December 9, 2023 in Guiyang City, China. Adult male current smokers were enrolled. Partial least squares structural equation modelling was used to test the hypothesis. The multi-group analysis was used to determine the moderating effect of cigarette dependence, and the importance-performance map analysis was utilised to assess the importance and performance of rationalisations.

**Results:**

A total of 616 adult male current smokers were analysed, and they were divided into the low cigarette dependence group (*n* = 297) and the high cigarette dependence group (*n* = 319). Except for risk generalisation beliefs, smoking functional beliefs (H1: -β = 0.131, *P* < 0.01), social acceptability beliefs (H3: β = -0.258, *P* < 0.001), safe smoking beliefs (H4: β = -0.078, *P* < 0.05), self-exempting beliefs (H5: β = -0.244, *P* < 0.001), and quitting is harmful beliefs (H6: β = -0.148, *P* < 0.01) all had a significant positive influence on motivation. Cigarette dependence moderated the correlation between rationalisations and motivation. In the high-dependence group, the social acceptability beliefs and smoking functional beliefs were located in the “Concentrate Here” area. In the low-dependence group, the social acceptability beliefs were also situated in there.

**Conclusions:**

Social acceptability beliefs and smoking functional beliefs showed great potential and value for improvement among high-dependence smokers, while only social acceptability beliefs had great potential and value for improvement among low-dependence smokers. Addressing these beliefs will be helpful for smoking cessation. The multi-group analysis and the importance-performance map analysis technique have practical implications and can be expanded to other domains of health education and intervention practice.

**Supplementary Information:**

The online version contains supplementary material available at 10.1186/s12889-024-19295-y.

## Introduction

Tobacco control is a huge challenge in China. The China Adult Tobacco Survey (CATS) revealed that the smoking rate in 2018 was 50.5% among males and 2.1% among females [1]. Meanwhile, Chinese smokers appear to be less inclined to quit. Merely 16.2% of current smokers planned to quit within a year, while a mere 5.6% planned to quit within a month [1]. To develop effective cessation strategies to inspire smokers to quit, it is imperative to acquire a more profound comprehension of the quitting process and the variables that exert influence upon it. The process of cessation is commonly categorised into three different stages: intention to quit, attempts to quit, and successful quitting [2]. Intention to quit is recognised as a predictor of quit attempts [3–6]. Since desire, duty, and intention to quit are three different aspects of motivation to quit smoking, motivation includes intent to quit [6]. Intention is an independent predictor of quit attempts [6], thus, motivation can also predict them, as has been verified in past literature [7, 8]. Moreover, quit attempts are a positive predictor of successful quitting [4, 5]. Therefore, it can be concluded that motivation is the primary determinant of cessation behaviour. Hence, it is crucial to boost the motivation of smokers to cease smoking in order to effectively increase smoking cessation rates.

Encouraging smokers to quit is a challenging task. It is common for smokers to not quit, even though they know that smoking is harmful to their health. This inconsistency between an individual's beliefs and behaviours might lead to cognitive dissonance. Festinger [9] proposed that cognitive dissonance is psychologically uncomfortable and unpleasant, so it drives individuals to alleviate this state [9]. One way to reduce dissonance is to quit smoking, but this is difficult for smokers. Another way is to change perceptions of the effects of smoking through misinterpretation, denial, or distortion of facts, which may prove less challenging than quitting [10]. Self-exempting beliefs are recognised as manifestations of the latter cognitive dissonance-reduction strategy of smokers [11]. Self-exempting beliefs, also known as smoking rationalisation beliefs (SRB), are a huge barrier to quitting smoking [12]. SRB refers to a cognitive and psychological phenomenon in which individuals tend to believe that smoking is a reasonable or acceptable behaviour, even though they may be aware of the potential harms of smoking. Smokers shield themselves from personal responsibility and the criticism of others by developing rationalisation beliefs that justify their smoking habit, enabling them to persist in their behaviour [13]. A significant number of scholars conducted in-depth research on the types of these beliefs. In general, these beliefs mainly contained smokers' suspicion of the harms of smoking, self-exemption from the harms caused by smoking, normalisation of smoking behaviour, emphasis on the positive effects, the belief that the harm can be mitigated through appropriate and sensible methods, and the notion that quitting smoking can also be detrimental [14–20]. Due to the influence of inherent concepts and customs in traditional Chinese culture as well as the nationalisation and penetration of China's tobacco industry, Chinese smokers' rationalisations towards smoking are more abundant and complex [21]. Huang et al. [15] developed a six-dimensional smoking rationalisation beliefs scale for Chinese male smokers [15]. Among them, smoking functional beliefs, self-exempting beliefs, and risk generalisation beliefs were consistent with findings from smokers in Australia, the UK, Canada, and the US, suggesting that some rationalisations may be shared by smokers from different cultural backgrounds. In contrast, social acceptability beliefs, safe smoking beliefs, quitting is harmful beliefs, and one of the items in smoking functional beliefs (“smoking can reduce interpersonal distance and make social interaction easier”) were not common among western smokers and were specific to Chinese smokers [15]. Chinese society widely accepts men's smoking behaviour, viewing it as a significant aspect of social interactions. Offering and accepting cigarettes is a common social ritual that fosters friendship and trust among men [22]. Chinese smokers commonly hold misconceptions about safe smoking, believing that it is safe to smoke high-quality cigarettes or low-tar cigarettes, or not inhale smoke into the lungs. In addition, influenced by Chinese medicine theory, many Chinese smokers believe that a person who has smoked for many years has established an "equilibrium" in the body, and that quitting smoking will upset this equilibrium and lead to disease, and therefore quitting smoking is harmful [15].

Several studies demonstrated that rationalisations had a significant negative correlation with motivation to quit [17, 18, 23, 24]. This is the reason why conventional approaches to discouraging smoking by highlighting its detrimental impact fail to achieve effective outcomes [25]. However, rationalisations are generated by smokers to dissolve their cognitive dissonance and do not consistently represent smokers' long-term views. Once the path to resolving dissonance through rationalisations is cut off, quitting smoking becomes the only way for smokers to resolve their cognitive dissonance [9, 14]. Therefore, interventions that address smokers' rationalisations may be helpful for smoking cessation [10].

In addition, it is essential to consider cigarette dependence when designing smoking cessation interventions [5]. Previous research has illustrated that levels of cigarette dependence are associated with smoking cessation motivations and behaviours (e.g., quit success, quit attempts). One study found that rationalisations were significantly and negatively related to motivation when controlling for nicotine dependence [17]. Several studies indicated that smokers with low cigarette dependence were more inclined to succeed in quitting smoking [8], whereas those with high cigarette dependence were more likely to experience unsuccessful cessation attempts [7, 26]. Additional research conducted in China demonstrated that cigarette dependence had a more pronounced impact on attempts to quit smoking than on maintaining abstinence [4] and that attempts to quit were significantly associated with low cigarette dependence [27]. Smokers with different levels of nicotine dependence differ in psychological factors of motivation to quit. A study confirmed that most PMT variables were significantly linked with intention among low tobacco-dependent smokers, yet none promoted intention to quit among high-dependent smokers [5]. These findings suggested that the relationship between psychological factors and motivation was susceptible to cigarette dependence. Since rationalisations are also psychological factors, it can be inferred that smokers with different levels of cigarette dependence differ in the relationship between rationalisations and motivation to quit. However, because rationalisations contain multiple factors, differences between high and low cigarette dependent smokers in the relationship between specific rationalisations and motivation remain unknown. Cigarette dependence may moderate the negative relationship between rationalisations and motivation to quit smoking. In addition, for smokers with a certain level of cigarette dependence, which rationalisations should be the focus of attention and improvement was still unknown in previous research.

This study sought to answer the following questions based on the analyses: (1) Do rationalisations significantly associate motivation to stop smoking? (2) Does cigarette dependence moderate the relationships between rationalisations and motivation to stop smoking? (3) Which rationalisations should be focused on for different levels of cigarette dependence? This study used partial least squares structural equation modelling (PLS-SEM) to examine the influence of rationalisations on motivation to quit smoking, multigroup analysis (MGA) to test the moderating effect of cigarette dependence, and importance-performance map analysis (IPMA) to assess the importance and performance of rationalisations on motivation among smokers with varying cigarette dependence.

The research model employed the smoking rationalisation scale for male smokers in China, developed by Huang et al. [15]. The exogenous independent factors were smoking functional beliefs (SFB), risk generalisation beliefs (RGB), social acceptability beliefs (SAB), safe smoking beliefs (SSB), self-exempting beliefs (SEB), and quitting is harmful beliefs (QHB). The endogenous dependent variable was motivation to stop smoking (MTSS). Hypotheses for study include:

H1: SFB has a negative correlation with MTSS.

H2: RGB has a negative correlation with MTSS.

H3: SAB has a negative correlation with MTSS.

H4: SSB has a negative correlation with MTSS.

H5: SEB has a negative correlation with MTSS.

H6: QHB has a negative correlation with MTSS.

H7: Cigarette dependence moderates the correlation between SRB and MTSS.

## Methods

### Participants and procedure

The participants were recruited from Guiyang City, the administrative hub of Guizhou Province. Situated in southwestern China, Guizhou Province is an economically disadvantaged province and a major producer of tobacco. Guizhou Province is beset by a severe smoking epidemic. The prevalence of smoking among individuals aged 15 and above in Guizhou has exceeded 30%, ranking it as the second highest in China [28]. Personal tobacco consumption exceeds 2,100 yuan, 60% above the national average [29].

The survey targeted adult male current smokers. The prevalence of smoking among Chinese women is only 2.1% [1], making research recruitment difficult. Compared with minor smokers, adult smokers are more likely to develop stable smoking habits and rationalisations to resist stopping [17]. Therefore, female smokers and minor smokers were excluded. According to the definition by the World Health Organization (WHO), smokers refer to those who have continuously or cumulatively smoked for six months or more in their lifetime. Furthermore, current smokers are defined as individuals who have smoked at least once in the last month [30, 31]. Based on previous studies using the Chinese Male Smoking Rationalisation Scale [12, 15, 19, 20], this study's survey subjects were required to be 18 or older, male, have smoked more than 100 cigarettes in their lifetime, and smoke at least once in the past month.

We collected questionnaires by conducting on-site interception visits in densely populated main streets, large communities, and urban parks in Guiyang from November 19 to December 9, 2023. Adult males were randomly intercepted and asked three questions, namely, "Do you smoke?" "Have you ever smoked 100 cigarettes in your life?" and "Have you ever smoked at least once in the last month?" If they answered yes to all three questions, they were asked to scan the QR code with their smartphone to complete the questionnaire, a way to ensure participants would fully complete the questionnaire. For participants with reading difficulties, impaired vision, or limited smartphone access for online surveys, the investigator verbally presented the questions and impartially recorded their responses on a paper questionnaire. Participants received five yuan as a reward. The Ethics Review Committee of Guizhou Medical University approved this study, with approval number 2022–297. Prior to participants completing the questionnaire, the investigator thoroughly explained the survey's objectives, emphasized the anonymity and confidentiality of individual survey results, and obtained informed consent from all participants through signature on a consent form.

### Measures

#### Smoking rationalisation beliefs

The study used the Chinese Male Smoking Rationalisation Scale developed by Xinyuan Huang et al. [15], which considered Chinese social and cultural aspects and had good validity and reliability. Table S1 displays all scale items. All survey questions utilised a 5-point Likert scale ranging from 1 (strongly disagree) to 5 (strongly agree). However, for future IPMA, all model indicators must have the same scale direction [32]. This means that a positive correlation between SRB and MTSS needs to be established in the model. The higher the score, the better the result, indicating that SRB is weaker. Therefore, it is very necessary to change the direction of the SRB scale when conducting IPMA, ranging from 1 (strongly agree) to 5 (strongly disagree).

#### Motivation to stop smoking

This study used the motivation to stop smoking scale developed by D. Kotz et al. [33]. The MTSS comprises a single item with seven response categories, ranging from 1 (indicating the absence of any belief, desire, or intention) to 7 (representing the strongest desire and short-term intention) [34, 35]. The MTSS accurately forecasts cessation efforts and assesses motivation [33].

#### The Fagerström Test for Cigarette Dependence

The widely utilised Fagerström Test for Cigarette Dependence (FTCD) (renamed from the Fagerström Test for Nicotine Dependence, FTND) was employed in the study [36–38]. It combines six items with a sum score from 0 (lowest) to 10 (highest dependence) [39]. The optimal threshold score for the FTCD to screen for cigarette dependence is 4 [40], which means that smokers with scores from 0 to 4 have low cigarette dependence, while smokers with scores from 5 to 10 have high cigarette dependence.

### Data analysis

#### Data cleaning and exclusion

The survey received 790 questionnaires. Several questions in the questionnaire were used to further confirm whether the respondents met the inclusion criteria. Those who did not meet the criteria were excluded. Before conducting MGA, it is critical to assess the quality of the data and identify the existence of a straight-lining pattern [41]. Therefore, response patterns consisting solely of "1" or "5" (representing the ultimate response) or "3" (representing the intermediate response) were eliminated, leaving 616 valid samples in the end.

#### Partial least squares analysis

This study employed the PLS-SEM method and the SmartPLS 4 software [42] to construct, estimate, and assess conceptual models. There are reasons for using PLS-SEM. Firstly, unlike traditional CB-SEM, which prioritises model fitting [43], PLS-SEM prioritises the optimisation of endogenous structural predictions [44], enabling researchers to assess the predictive quality [45]. Thus, PLS-SEM is advantageous for estimating structural models that elucidate key target structures [46, 47]. Given that the main aim of this study was to forecast the motivation of smokers based on rationalisations rather than testing a theory, the PLS-SEM method was selected. Secondly, unlike CB-SEM, which has uncertain factor scores [43, 48], PLS-SEM offers the fixed latent variable scores needed for IPMA. The IPMA assesses the total effect of the predecessor constructs on the target construct by comparing it to the average latent variable scores indicating their performance [32, 44, 46]. Furthermore, PLS-SEM is appropriate for situations involving limited sample sizes and non-probability sampling, which are advantages that CB-SEM lacks [49–51].

In this study, SmartPLS 4 software was used to run the PLS-SEM algorithm to estimate the reliability and validity of the measurement model and the explanation power of the target construct. Then, 10,000 times of bootstrapping were run to estimate the path coefficients and significance. Any insignificant path is excluded. The G*Power software was used to perform efficacy analyses and estimate the required minimum sample size for each group [52, 53]. The measurement invariance of composite models (MICOM) procedure was then conducted since it served as a prerequisite for MGA. If the results showed evidence of partial or full measurement invariance, the MGA could be performed [54]. MGA was then used to evaluate the moderating effects of multiple relationships in the model [41]. After that, an IPMA was run to cross-check the group results and develop specific conclusions for each group [55–57]. The objective is to ascertain antecedents that possess substantially high importance for the target construct while simultaneously exhibiting a comparatively low level of performance [32]. The current study determined the importance of SRB for MTSS and the performance of these beliefs in each group. It also helped us discover key but underperforming beliefs in each group.

## Results

### Sample profile and groups

The survey retained 616 of 790 questionnaires. The sample had the most people aged 25 to 34 (*n* = 209, 33.9%), followed by 35 to 44 (*n* = 191, 31.0%), and 18 to 24 (*n* = 124, 20.1%). Most people had bachelor or college degrees (*n* = 332, 53.9%), while a significant portion had high school or vocational school degrees (*n* = 198, 32.1%). Most were workers or enterprise personnel (*n* = 244, 39.6%), followed by self-employed individuals (*n* = 112, 18.2%) and civil servants or public institution personnel (*n* = 96, 15.6%). Most smokers (54.1%, *n* = 333) initiated smoking between 18 and 24, while 27.3% (*n* = 168) started between 12 and 17. SFB, RGB, SAB, SSB, SEB, and QHB had mean values of 3.78, 3.66, 3.52, 3.20, 3.14, and 3.27 (on a scale of 1 to 5, with 1 being extreme disagreement and 5 being estreme agreement). The average motivation to quit smoking was 3.06. We divided cigarette dependence into a low dependence group (*n* = 297) and a high dependence group (*n* = 319) based on previous studies [38, 40].

### Measurement model assessment

In the first analysis step, we evaluated the measurement model results of the total sample [58]. Table 1 displays the standardised factor loadings and their T-values for the measurement indicators, along with the Cronbach's alpha, composite reliability, and average variance extracted (AVE) values for the constructs. The findings indicate that the standardised factor loadings for each indicator exceed 0.694 and are significant (significance can be obtained from bootstrapping). Additionally, with the minimum value of the Cronbach's alpha value and the composite reliability being 0.659 and 0.673, respectively, all values surpass the threshold of 0.6 [44]. The average variance extracted (AVE) exceeds 0.595, which satisfies the requirement of being greater than 0.5 [44, 59]. The measurement model demonstrates strong convergent validity.

**Table 1:** Reliability and convergent validity

Constructs	Indicators	Standardized factor loading	T-value	Cronbach's alpha	Composite reliability	Average variance extracted (AVE)
Smoking functional beliefs (SFB)	SFB 1	0.842	53.085	0.840	0.849	0.610
	SFB 2	0.821	48.529			
	SFB 3	0.766	30.712			
	SFB 4	0.740	36.185			
	SFB 5	0.730	21.787			
Risk generalization beliefs (RGB)	RGB 1	0.800	33.208	0.659	0.673	0.595
	RGB 2	0.814	40.734			
	RGB 3	0.694	18.796			
Social acceptability beliefs (SAB)	SAB 1	0.852	61.236	0.891	0.895	0.650
	SAB2	0.797	34.891			
	SAB3	0.832	52.089			
	SAB4	0.847	60.831			
	SAB 5	0.703	25.208			
	SAB 6	0.797	41.700			
Safe smoking beliefs (SSB)	SSB1	0.787	33.921	0.848	0.858	0.686
	SSB2	0.842	48.531			
	SSB3	0.845	55.602			
	SSB4	0.836	63.362			
Self-exempting beliefs (SEB)	SEB1	0.848	54.318	0.903	0.904	0.720
	SEB2	0.860	66.006			
	SEB3	0.858	58.258			
	SEB4	0.836	47.433			
	SEB5	0.842	49.504			
Quitting is harmful beliefs (QHB)	QHB1	0.845	48.219	0.831	0.834	0.747
	QHB2	0.866	60.147			
	QHB3	0.881	69.505			
MTSS	MTSS1	1	n/a	n/a	n/a	n/a

Furthermore, according to the Fornell-Larcker criterion matrix [60], if the latent variables are truly distinct constructs, their respective measurement indicators should have stronger correlations with their own latent variable than with other latent variables. Thus, if the square root of the AVE for each construct is higher than the highest correlation between that construct and any other construct in the model, then the model exhibits discriminant validity [44]. Table 2 demonstrates that each diagonal value is greater than the values below it, indicating successful discriminant validity.

**Table 2:** Fornell-Larcker criteria matrix

	SFB	RGB	SAB	SSB	SEB	QHB	MTSS
SFB	0.781	-	-	-	-	-	-
RGB	0.620	0.771	-	-	-	-	-
SAB	0.624	0.701	0.806	-	-	-	-
SSB	0.439	0.460	0.643	0.828	-	-	-
SEB	0.521	0.554	0.736	0.713	0.849	-	-
QHB	0.533	0.547	0.699	0.618	0.786	0.864	-
MTSS	-0.550	-0.544	-0.694	-0.581	-0.691	-0.655	1

The diagonal numbers in the matrix represent the square roots of the AVEs for the constructs. The other numbers represent the correlations between the constructs

### Structural model assessment

**Fig. 1:**
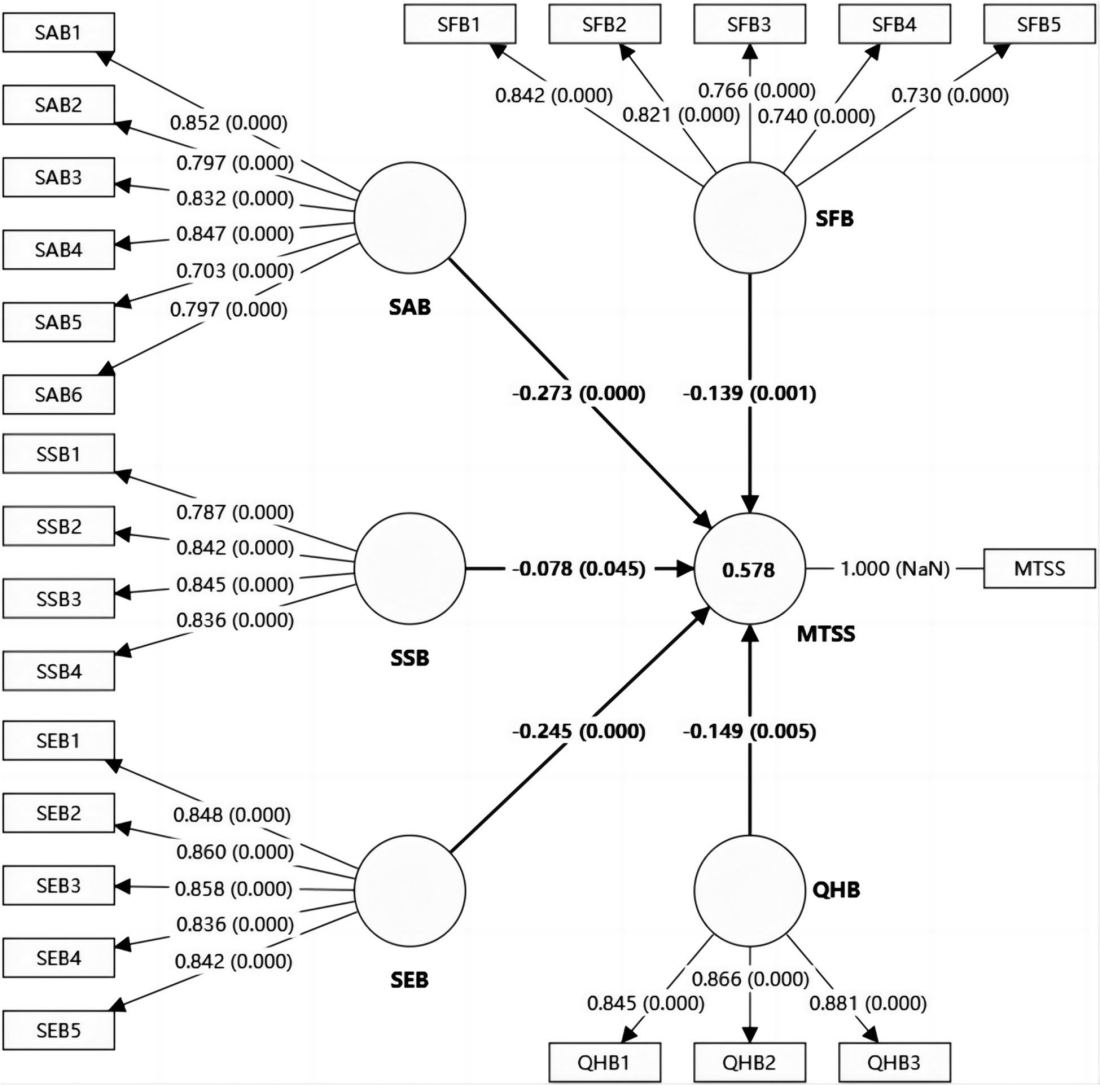
Bootstrapping results. Note: SFB = Smoking functional beliefs; SAB = Social acceptability beliefs; SSB = Safe smoking beliefs; SEB = Self-exempting beliefs; QHB = Quitting is harmful beliefs; MTSS = Motivation to stop smoking

The second analysis step was to calculate the PLS path model [61]. Table 3 displays the standardised path coefficients, T-values, and their significance for the inner model. The results indicate that although the impact of RGB (H2: β = -0.030, *P* > 0.05) on MTSS is not significant, SFB (H1: -β = 0.131, *P* < 0.01), SAB (H3: β = -0.258, *P* < 0.001), SSB (H4: β = -0.078, *P* < 0.05), SEB (H5: β = -0.244, *P* < 0.001), and QHB (H6: β = -0.148, *P* < 0.01) all have a negative and significant influence on MTSS. This indicates that the stronger these five rationalisations, the weaker the motivation to quit smoking. Moreover, the *R*^2^ value of MTSS is 0.578, indicating that all rationalisations explain 57.8% of the variance of MTSS [53]. Hair suggested that an *R*^2^ value of 0.5 indicates moderate explanatory power [53]. For consequent MGA and IPMA, the insignificant path H2 and RGB construct were removed, and the 1000-time bootstrapping results after removing them are shown in Fig. 1.

**Table 3:** Summary of hypotheses testing results

Hypothesis	Path	Standardized path coefficient	T-value	Supported
H1	SFB → MTSS	-0.131**	3.016	Yes
H2	RGB → MTSS	-0.030	0.647	No
H3	SAB → MTSS	-0.258***	4.780	Yes
H4	SSB → MTSS	-0.078*	2.021	Yes
H5	SEB → MTSS	-0.244***	4.205	Yes
H6	QHB → MTSS	-0.148**	2.783	Yes

^*^P-value < 0.05

^**^P-value < 0.01

^***^P-value < 0.001

### Multigroup analysis (MGA)

Based on a power analysis using G * Power software [52, 62], with 6 predictive variables, each group needs 98 observations to reach effect size f^2^ values of 0.15 with a significance level of 5% and a statistical power of 80%. Thus, both groups' sample sizes satisfied this criterion.

In order to guarantee the accuracy and reliability of the MGA results, Henseler et al. [54] introduced the MICOM procedure, which included configural invariance, compositional invariance, and the equality of composite mean values and variances [54]. The configural invariance was established throughout data processing. This study then performed permutation multigroup analysis in SmartPLS and set 5000 permutations for one-tailed testing at a significance level of 0.05. As shown in Table 4, the value of the 5% quantile is no more than correlation values for all constructs, indicating compositional invariance is established. Although the composite's mean construct values do not fall within the 95% confidence interval, its variance values do. The results indicated partial measurement variance [41, 54].

**Table 4:** The compositional invariance and the equality of composite mean values and variances

Composite	Correlation value (= 1)	5% quantile	Compositional invariance?
SFB	1.000	0.997	Yes
SAB	1.000	0.999	Yes
SSB	0.998	0.998	Yes
SEB	1.000	0.999	Yes
QHB	0.999	0.999	Yes
MTSS	1.000	1.000	Yes
**Composite**	**Difference of the composite's mean value (= 0)**	**95% confidence interval**	**Equal mean values?**
SFB	0.650	[-0.133; 0.132]	No
SAB	0.695	[-0.135; 0.132]	No
SSB	0.575	[-0.135; 0.133]	No
SEB	0.672	[-0.135; 0.130]	No
QHB	0.727	[-0.131; 0.135]	No
MTSS	-0.661	[-0.135; 0.133]	No
**Composite**	**Difference of the composite's variance value (= 0)**	**95% confidence interval**	**Equal variances?**
SFB	-0.017	[-0.254; 0.254]	Yes
SAB	-0.045	[-0.186; 0.186]	Yes
SSB	0.134	[-0.170; 0.167]	Yes
SEB	0.049	[-0.164; 0.161]	Yes
QHB	-0.071	[-0.162; 0.158]	Yes
MTSS	-0.117	[-0.172; 0.171]	Yes

Subsequently, the study performed MGA to examine the two groups, as indicated in Table 5. Within the high-dependence group, all beliefs except SSB and QHB negatively and significantly affect MTSS. In the other group, all beliefs except SSB demonstrate a negative and significant influence on MTSS. Moreover, one difference in path coefficients between the high dependence group and the low dependence group is significant (H6: Diff = 0.255, *P* < 0.01), indicating it is not invariant. Consequently, the MGA findings supported hypothesis 7, suggesting that cigarette dependence moderates the connection between SRB and MTSS. Additionally, the *R*^2^ value of the MTSS is 0.490 in the high-dependence group and 0.582 in the low-dependence group. Both suggest moderate explanatory power.

**Table 5:** Multi-group analysis result

Hypothesis	Path	Standardized path (high-dependence)	T-value (high-dependence)	Standardized path (low-dependence)	T-value (low-dependence)	Diff(high-low)	Invariant
H1	SFB → MTSS	-0.177**	2.762	-0.099*	2.194	-0.078	Yes
H3	SAB → MTSS	-0.265***	3.369	-0.273***	4.278	0.009	Yes
H4	SSB → MTSS	-0.061	1.24	-0.098	1.470	0.036	Yes
H5	SEB → MTSS	-0.315***	3.883	-0.153*	1.969	-0.162	Yes
H6	QHB → MTSS	-0.013	0.188	-0.269***	3.777	0.255**	No

^*^P-value < 0.05

^**^P-value < 0.01

^***^P-value < 0.001

### Importance-performance map analysis (IPMA)

**Fig. 2:**
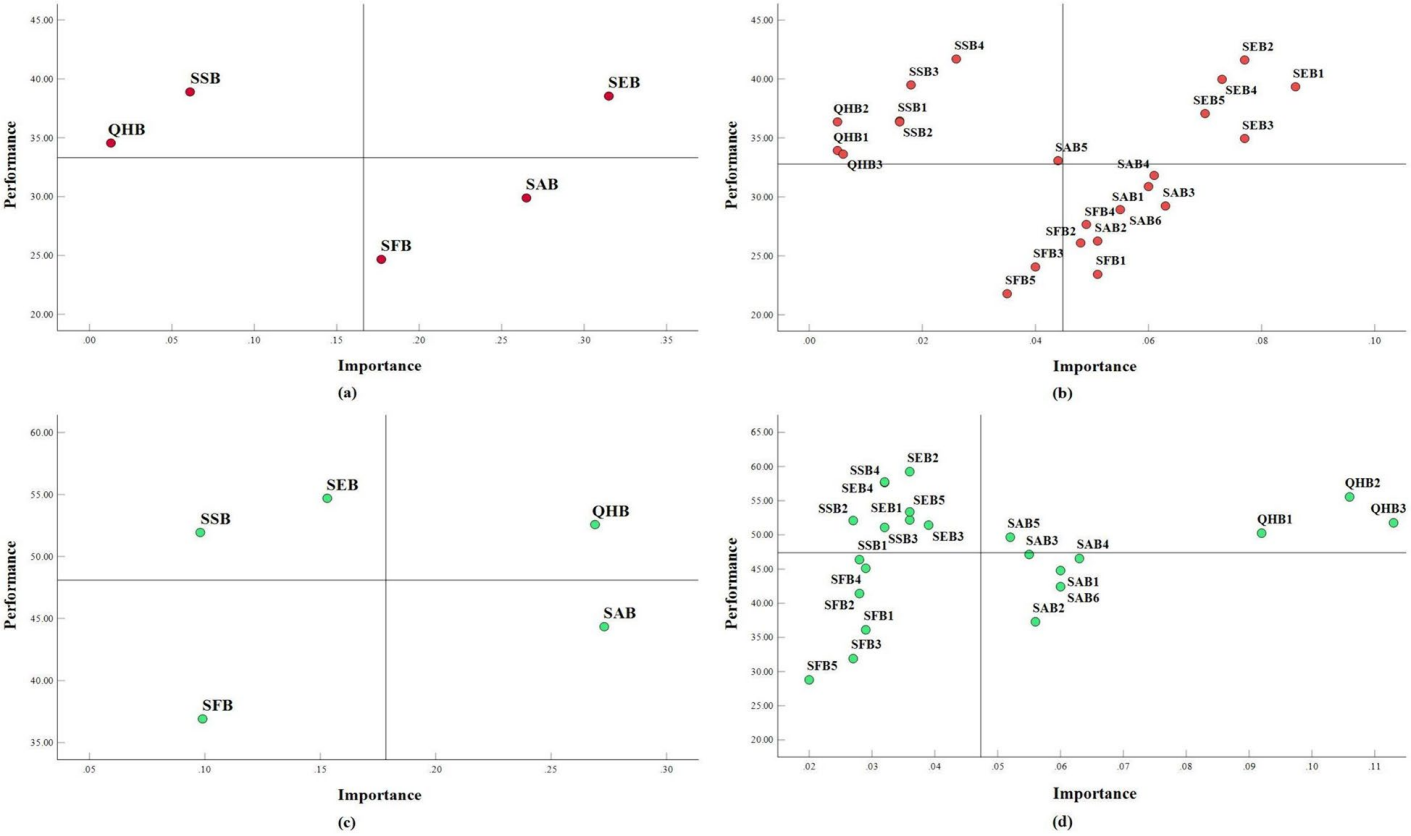
Importance-performance map of motivation. Note: SFB = Smoking functional beliefs; SAB = Social acceptability beliefs; SSB = Safe smoking beliefs; SEB = Self-exempting beliefs; QHB = Quitting is harmful beliefs

To conduct IPMA later, all model indicators must have the same scale direction [32], and thus the direction of the SRB scale was changed, ranging from 1 (strongly agree) to 5 (strongly disagree). IPMA was then conducted to assess SRB performance and their importance in MTSS in two groups. Figure 2 displays the importance-performance maps of five SRB constructs and 23 associated indicators for both groups. The X-axis in the importance-performance map represents the importance of the constructs or indicators to the target construct, and the Y-axis represents the performance of the constructs or indicators on the mean latent variable scores after rescaling to a range of 0 to 100 [32]. To enhance orientation, a vertical mean line and a horizontal mean line divided the coordinate system into four quadrants. The upper-right quadrant demonstrates higher importance and performance above average (i.e., “Keep Up the Good Work”). This means that the beliefs within this quadrant are crucial to the motivation to stop smoking and are performing well (indicating weak beliefs), hence, it is recommended to maintain them. Conversely, the upper-left quadrant exhibits lower importance but higher performance than average (i.e., “Possible Overkill”). It suggests that excessive effort has been put into reducing these beliefs, which are not particularly important for motivation. Therefore, it is advisable to maintain an average level of attention towards these beliefs. Below-average importance and performance are in the lower-left quadrant (i.e., “Low Priority”). These beliefs are the least important for motivation and poorly performed (indicating strong beliefs), thus the least amount of attention is recommended for these beliefs. Lastly, the lower-right quadrant reveals higher importance but lower performance than average (i.e., “Concentrate Here”). These beliefs are critical for enhancing motivation to quit smoking but are currently underperforming (indicating strong beliefs). Therefore, special attention is strongly recommended to address these beliefs [63, 64].

Panel (a) and (b) of Fig. 2 display the five SRB constructs and 23 associated indicators for the high-dependence group, respectively. The SEB construct and all associated indicators are located in the upper-right quadrant. Similarly, the SSB and QHB constructs, along with all associated indicators, are situated in the upper-left quadrant. On the other hand, the SAB and SFB constructs, along with the majority of indicators, are located in the lower-right quadrant. Panel (c) and (d) of Fig. 2 show the constructs and indicators of the low-dependence group, respectively. The QHB construct and all associated indicators are located in the upper-right quadrant. Similarly, the SSB and SEB constructs, along with the majority of associated indicators, are situated in the upper-left quadrant. On the other hand, the SFB constructs, along with all the associated indicators, are located in the lower-left quadrant, while the SAB constructs and the majority of the associated indicators are situated in the lower-right quadrant.

## Discussion

Our analysis demonstrated that the model exhibited strong performance in terms of reliability, convergent validity, and discriminant validity. In this study, the total variance of the explanation for motivation to quit smoking as an endogenous variable in the total sample was 57.8%, demonstrating good explanatory power. This finding suggested that the rationalisations of smokers can greatly predict their motivation to quit smoking, thus verifying the empirical results of previous studies [12].

For the first question, the study found that risk generalisation beliefs had no significant association with motivation to stop smoking, unlike previous studies that found all six types of beliefs negatively associated with intention [12]. Similar to the jungle beliefs previously proposed by Oakes et al. [15, 18], risk generalisation beliefs mean that the harms of smoking do not seem as great when compared with other harms in life. For example, "Air pollution, food safety, and life stress are much more dangerous to health than smoking." One study also confirmed the weak association between jungle beliefs and the motivation to quit smoking [19, 20]. Since the widespread dissemination of information about the detrimental effects of tobacco through various media channels has ingrained a strong perception in the public, smokers are not convinced that the harms caused by smoking are less severe than other forms of harm. Conversely, social acceptability beliefs had the most strongly negative influence on motivation, which had been confirmed in past studies [65]. Compared with smokers in Western countries, social acceptability beliefs are specific to Chinese smokers [4, 15]. In Chinese smoking culture, cigarettes symbolise prestige and achievement, and offering and accepting them is common in social interactions [22]. Despite growing public health awareness and tougher tobacco control, altering this deeply ingrained smoking culture within a short timeframe is challenging [66]. Furthermore, given that numerous prominent male individuals, including celebrities, physicians, educators, and others, engage in smoking, it is widely acknowledged and even "anticipated" that men will partake in this habit, thus obviating the necessity for them to cease [12]. Similar to prior studies, self-exempting beliefs also had a notable relationship with motivation [67]. These beliefs underestimate an individual's personal susceptibility to disease, foster a belief in personal invulnerability to the negative effects of smoking, and exhibit an unrealistic optimism about one's health.

Regarding the second research question, the present study found that cigarette dependence moderated the correlation between rationalisations and motivation to quit through MGA. Surprisingly, quitting is harmful beliefs did not have a significant impact on motivation to stop smoking in the high-dependence group, while it had a significantly negative effect in the low-dependence group. This discovery was unexpected. Smokers in the high-dependence group have developed a strong reliance on cigarettes and may suffer intense withdrawal symptoms including anxiety, irritability, difficulty concentrating, and weight gain, which makes them create the perception that quitting is detrimental and makes them reluctance to quit. In contrast, the low-dependence smokers were less likely to experience these symptoms, therefore, they were less likely to believe quitting smoking was harmful and less likely to refuse to quit. This result was difficult to explain, but it may be related to the influence of several other rationalisations on the target construct. Within the high-dependence group, the association between quitting is harmful beliefs and motivation to quit is less strong when compared to smoking functional beliefs, social acceptability beliefs, and self-exempting beliefs. Conversely, within the low-dependence group, quitting is harmful beliefs exhibit a stronger correlation with motivation to stop smoking when compared to smoking functional beliefs, safe smoking beliefs, and self-exempting beliefs. It was also worth mentioning that the influence of safe smoking beliefs on the motivation to stop smoking was not significant in either group. However, they had a weaker but still significant negative correlation in the total sample. This is due to the fact that as the sample size increases, the distribution of random errors becomes more stable, and the relationship between variables is more likely to be significant.

In order to address the third question, this study conducted an IPMA to offer more information about the specific rationalisations that require attention at different levels of cigarette dependence. The constructs and indicators located in the “Keep Up the Good Work” area (upper-right quadrant) exhibit great importance and a high level of performance (indicating weak beliefs) [63]. The self-exempting beliefs of high-dependence smokers were located there, suggesting that these beliefs were the primary motivators for high-dependence smokers to quit smoking. It also showed that these weak self-exempting beliefs performed well, leaving no room for further improvement. The primary cause of this phenomenon is that smoking cessation initiatives in China have predominantly emphasised the hazards associated with smoking, thereby making it improbable for high-dependence smokers to cultivate such beliefs that they would self-exempt from the harms of smoking. Furthermore, among the low-dependence smokers, quitting is harmful beliefs located in the same area, indicating that they were less likely to hold the belief that quitting smoking was detrimental to their health. It suggested that the low-dependence smokers were primarily motivated by their quitting is harmful beliefs, and they had no room for further improvement. Therefore, it was recommended to maintain the self-exempting beliefs of high-dependence smokers, and quitting is harmful beliefs of low-dependence smokers.

The constructs and indicators located in the "Concentrate Here" area (lower-right quadrant) are important but perform poorly (indicating strong beliefs). It offered the greatest potential and value for improvement [63]. Smoking functional beliefs of high-dependence smokers located there. This implied that the high-dependence smokers were more inclined to acknowledge the numerous advantages of smoking, such as alleviating fatigue, enhancing mental alertness, and facilitating social interactions. Consequently, smoking functional beliefs served as the primary obstacles preventing high-dependence smokers from quitting, and they hold great potential and requirement for improvement. It was important to note that the three strongest smoking functional beliefs among them—“smoking can eliminate fatigue and be refreshing” (SFB1), “smoking is good for inspiration and active thinking” (SFB2), and “smoking is a good way to kill time” (SFB4)—required the most improvement. Health education on smoking cessation can be provided to high-dependence smokers, allowing them to understand that although nicotine in tobacco briefly stimulates the nervous system and refreshes the mind, long-term smoking can actually lead to negative effects such as fatigue, lack of energy, lack of concentration, and decreased memory. Instead, a healthy lifestyle and good work habits are the keys. In addition, they should be encouraged to pursue healthier activities like reading, exercising, and socializing, which can enhance their quality of life, and lessen their reliance on tobacco.

It was also significant to point out that, regardless of whether it's the high-dependence group or the low-dependence group, social acceptability beliefs were all located there. This suggested that social norms were crucial in the formation of rationalisations among Chinese male smokers, which was consistent with the research findings of Lee et al. [68]. They found that smokers in Thailand, where strict tobacco control policies were in place, experienced more negative social norms about smoking, leading to lower levels of rationalisation. Conversely, in Malaysia, where tobacco control policies were still in their infancy, there were fewer negative social norms about smoking, resulting in more prevalent rationalisations [68]. It also revealed that in China, the social acceptability of smoking remained high, indicating that there was still much room for improvement in the establishment of social norms related to tobacco control. Therefore, future tobacco control policies in China should focus more on strengthening social norms to discourage smoking, thereby diminishing social tolerance towards smoking and fostering the widespread adoption of healthy lifestyles. Specifically, the three strongest social acceptability beliefs that require the most improvement were “smoking is pretty normal for men” (SAB2), “smoking is a part of my lifestyle that others can't interfere with”(SAB6), and “many famous people smoke, so it is normal to smoke” (SAB1). These three beliefs stemmed from traditional gender identity, the quest for independence and autonomy, and imitation and conformity, respectively [22, 66]. In order to improve these beliefs, the stereotypical association between smoking and masculinity should be broken, emphasising the importance of healthy lifestyles. Secondly, through health education, we can enable smokers to fully understand the infringement that their smoking behaviour poses on the health rights of others. Ultimately, it is imperative to implement strict regulations on the speech and behaviour of prominent people, with the aim of directing the public towards developing an accurate perception of smoking and thereby reducing the societal prestige associated with it.

### Limitations and implications

There were several limitations to our study. The study sample was restricted to adult male smokers in Guiyang City, resulting in limited generalizability. It adopted the smoking rationalisation scale suitable for Chinese male smokers, which prevented its extension to other countries. Furthermore, due to the cross-sectional study, it was impossible to monitor the rationalisations changes and smoking cessation status of smokers. Additionally, there might be interrelationships between beliefs and motivation. Rationalisations can affect motivation to quit, but motivation might also affect beliefs since a substantial reduction in such beliefs accompanies successful quitting. The cross-sectional design was unable to distinguish this, which was another limitation.

Despite some limitations, our findings had some theoretical and practical implications. It provided a useful supplement to the existing theoretical basis of rationalisations. It also extended the application of MGA and IPMA to assess the importance and performance of rationalisations on motivation to stop smoking in both the high and low cigarette dependence groups. This particular aspect had not been explored in prior research. The results of the IPMA classified rationalisations into four categories, which identified the key beliefs that need to be paid attention to, and indicated the focus of smoking cessation education and interventions. This technique had the potential to be expanded to other domains of health education and intervention practice.

## Conclusions

Smoking rationalisation beliefs significantly influenced motivation to stop smoking, except for risk generalisation beliefs. Cigarette dependence moderated the relationships between rationalisations and motivation. Social acceptability beliefs and smoking functional beliefs should be focused on for high-dependence smokers, while only social acceptability beliefs should be focused on for low-dependence smokers. Addressing these beliefs will be helpful for smoking cessation.

### Supplementary Information

Supplementary Material 1.

Supplementary Material 2.

## Data Availability

Data is provided within the manuscript or supplementary information files.

## Abbreviations

SRB: Smoking rationalisation beliefs
SFB: Smoking functional beliefs
RGB: Risk generalisation beliefs
SAB: Social acceptability beliefs
SSB: Safe smoking beliefs
SEB: Self-exempting beliefs
QHB: Quitting is harmful beliefs
MTSS: Motivation to stop smoking
PLS-SEM: Partial least squares structural equation modelling
MGA: Multigroup analysis
IPMA: Importance-performance map analysis
