# Supplementary material for: Breaking down barriers: rationalisations and motivation to stop among Chinese male smokers under cigarette dependence
Source: BMC Public Health. 2024 Jul 8;24:1812. doi: 10.1186/s12889-024-19295-y (PMC11229221; doi:10.1186/s12889-024-19295-y)
Supplement: Supplementary file 1 — Supplementary Material 1. [file 12889_2024_19295_MOESM1_ESM.docx]

Supporting Information

Table S1. Measurement items of constructs

| **Construct** | **Source** | **Indicator code** | **Measurement item** |
| --- | --- | --- | --- |
| Smoking functional beliefs (SFB) | Xinyuan Huang et al. (2020) (1) | SFB1 | Smoking can eliminate fatigue and be refreshing. |
|  |  | SFB2 | Smoking is good for inspiration and active thinking. |
|  |  | SFB3 | Smoking can reduce interpersonal distance and make social interaction easier. |
|  |  | SFB4 | Smoking is a good way to kill time. |
|  |  | SFB5 | Smoking can relieve tension and stress. |
| Risk generalization beliefs (RGB) | Xinyuan Huang et al. (2020) (1) | RGB1 | Air pollution, food safety, and life stress are much more dangerous to health than smoking. |
|  |  | RGB2 | If smoking was so bad for health, the government would have banned tobacco sales. |
|  |  | RGB3 | A lot of non-smokers also get lung cancer. |
| Social acceptability beliefs (SAB) | Xinyuan Huang et al. (2020) (1) | SAB1 | Many famous people smoke, so it is normal to smoke. |
|  |  | SAB2 | Smoking is pretty normal for men. |
|  |  | SAB3 | There are so many smokers in society that it’s hard for one to be different. |
|  |  | SAB4 | Lots of doctors smoke, so it’s unconvincing for them to persuade me to quit. |
|  |  | SAB5 | I will consider quitting smoking only if the government closes the tobacco factory. |
|  |  | SAB6 | Smoking is a part of my lifestyle that others can’t interfere with. |
| Safe smoking beliefs (SSB) | Xinyuan Huang et al. (2020) (1) | SSB1 | Low-tar cigarettes can reduce the harms of smoking/is less harmful. |
|  |  | SSB 2 | If you don’t inhale the smoke into the lungs, the harm is minimized. |
|  |  | SSB 3 | People like me who do not smoke many cigarettes are not at risk of smoking health problems. |
|  |  | SSB4 | It’s safe to smoke high-quality cigarettes. |
| Self-exempting beliefs (SEB) | Xinyuan Huang et al. (2020) (1) | SEB1 | I have not experienced any harm to my health. |
|  |  | SEB2 | I think I may have genes which protect me from the harms of smoking. |
|  |  | SEB3 | Smoking is not always bad for you because many smokers live long lives while many non-smokers don’t. |
|  |  | SEB4 | There is still insufficient medical evidence to prove that smoking is harmful. |
|  |  | SEB5 | The fact that I can still smoke means my health status is not bad. |
| Quitting is harmful beliefs (QHB) | Xinyuan Huang et al. (2020) (1) | QHB1 | If you have smoked for a long time, the body has adapted to smoking and reached a balance, so quitting will lead to illness. |
|  |  | QHB2 | After quitting smoking, I will gain weight, which is also harmful to my health. |
|  |  | QHB3 | If you try to quit and fail, you will smoke even more than before, so it is better not to quit. |
| Motivation to stop smoking (MTSS) | D. Kotz et al.(2013) (2) | MTSS1 | Question: Which of the following describes you?  Items:(1) I don’t want to stop smoking (1 point);  (2) I think I should stop smoking but don’t really want to (2 points);  (3) I want to stop smoking but haven’t thought about when (3 points);  (4) I REALLY want to stop smoking but I don’t know when I will (4 points);  (5) I want to stop smoking and hope to soon (5 points);  (6) I REALLY want to stop smoking and intend to in the next 3 months (6 points);  (7) I REALLY want to stop smoking and intend to in the next month (7 points). |
| the Fagerström Test for Cigarette Dependence  (FTCD) | Heatherton TF et al.(1991) (3) | FTCD1 | Question 1: How soon after you wake up do you smoke your first cigarette?  Items: (1) Within 5 minutes (3 points); (2) 6-30 minutes (2 points);  (3) 31-60 minutes (1 point); (4) After 60 minutes (0 point)  Question 2: Do you find it difficult to refrain from smoking in places where it is forbidden e.g. in church, at the library, in cinema, etc.?  Items: (1) Yes (1 point); (2) No (0 point)    Question 3: Which cigarette would you hate most to give up?  Items: (1) The first one in the morning (1 point); (2) All thers (0 point)    Question 4: How many cigarettes per day do you smoke?  Items: (1) 10 or less (0 point); (2) 11-20 (1 point)  (3) 21-30 (2 points); (4) 31 or more (3 points)  Question 5: Do you smoke more frequently during the first hours after waking than during the rest of the day?  Items: (1) Yes (1 point); (2) No (0 point)  Question 6: Do you smoke if you are so ill that you are in bed most of the day?  Items: (1) Yes (1 point); (2) No (0 point) |

1. Huang X, Fu W, Zhang H, Li H, Li X, Yang Y, et al. Development and validation of a smoking rationalization scale for male smokers in China. J Health Psychol [Internet]. 2020 Mar [cited 2023 Apr 5];25(4):472–89. Available from: http://journals.sagepub.com/doi/10.1177/1359105317720276

2. Kotz D, Brown J, West R. Predictive validity of the Motivation To Stop Scale (MTSS): A single-item measure of motivation to stop smoking. Drug and Alcohol Dependence [Internet]. 2013 Feb [cited 2023 Oct 27];128(1–2):15–9. Available from: https://linkinghub.elsevier.com/retrieve/pii/S0376871612002864

3. Heatherton TF, Kozlowski LT, Frecker RC, Fagerström KO. The Fagerström Test for Nicotine Dependence: a revision of the Fagerström Tolerance Questionnaire. Br J Addict. 1991 Sep;86(9):1119–27.
